# Trends in suicide-related research in Australia

**DOI:** 10.1186/s13033-019-0335-2

**Published:** 2020-01-10

**Authors:** Marisa Schlichthorst, Lennart Reifels, Karolina Krysinska, Maria Ftanou, Anna Machlin, Jo Robinson, Jane Pirkis

**Affiliations:** 1 Centre for Mental Health, Melbourne School of Population and Global Health, The University of Melbourne, Carlton, 3010 Australia; 2 Orygen, The National Centre of Excellence in Youth Mental Health, Parkville, 3052 Australia

**Keywords:** Suicide, Research, Trends

## Abstract

**Background:**

Despite continuous research over the past 20 years in Australia there is still limited understanding of what works and what does not work in suicide prevention and where to invest research efforts that will help to expand this knowledge base. There is a recursive relationship between research activities, knowledge gain and the development of strategy and action plans as these in turn guide future decisions on research funding. In this context, the first step to continuous improvement in knowledge is to better understand where research has been invested in the past until now and where it has not.

**Methods:**

We conducted a study that collected data over two periods. The first data collection was done in 2006 for the period of 1999 to 2006 and the second data collection was in 2017 for the period from 2010 to 2017. This allowed us to examine changes in published suicide-related journal articles, and grants/fellowships funded between the two periods. Published articles and grants/fellowships were classified according to a pre-determined framework.

**Results:**

The number of suicide-related articles and grants/fellowships increased over the two periods. We noted shifts in the types of research that were funded and published, and in the emphasis that was given to different types of suicidal behavior, suicide methods, and settings. Research target groups showed a trend towards increasing diversification.

**Conclusions:**

Our findings help to identify current research priorities and inform where future priorities for suicide-related research in Australia lie by linking findings to other external data sources (population risk data, stakeholder consultations, national strategies and action plan documents).

## Background

Suicide continues to be a serious public health concern in Australia. The latest statistics show an increase from 11.0 per 100,000 in 2008 to 12.7 per 100,000 in 2017 [1]. Our knowledge of the epidemiology of suicide-related behaviors is quite strong, but there are still many gaps in our understanding of how to prevent suicide [2]. Past reviews of literature highlight that still little is known about what works in suicide prevention [2–6] and that different approaches work for different population groups under varying conditions. For example, limiting access to lethal means has been shown to reduce suicide risk, yet this finding is based on research on firearms and use of drugs [2]. Very little is known about how to prevent hanging, yet in Australia hanging is the most commonly used lethal suicide method [7]. Findings for specific high-risk population groups seem inconclusive and depend in part on the setting of delivery for the intervention, for example family interventions for suicidal youth show promising results while community and family interventions for adolescents with severe mental illness were not effective [2]. Drawing implications from current literature on effectiveness in suicide prevention strategies is therefore limited.

One of the reasons that our knowledge has not progressed further may be that research activities may have focused on a selected range of areas which may not create sufficient evidence upon which to progress suicide prevention efforts collectively. To date still little is known about whether researchers conduct the right type of studies, meaning those that help us to reduce suicide in the population; analyze suicidal behavior in all its facets; examine the most common or the most lethal suicide methods (which vary by country); focus on the groups for whom the burden of suicide is the greatest, or conduct studies in the settings that align with priorities of national strategies or reach the most vulnerable population groups. In Australia, high-risk population groups include for example young to middle aged people, Indigenous people, men, people with mental health problems or those who have history of suicide attempt [8, 9]. Therefore, it could be expected to see high research activity and investment in these areas. In conjunction with high-risk groups, studying specific settings and their use for effective intervention has also shown potential in reaching and impacting on vulnerable individuals. For example, improving (mental) health care settings can support suicide attempters; workplaces can help to target men as men are generally less inclined to seek help through the conventional health system [10, 11]; schools are able to reach adolescents and communities can engage in social support and early detection of risk especially in isolated population groups [12, 13].

Acknowledging that suicidal behavior is a complex and multifaceted issue that needs addressing in a number of different ways, we need to gain a better understanding of what kinds of studies are being conducted and if they are designed to inform questions about what works, for whom, and in what context to progress our knowledge with respect to suicide prevention. The first step in this direction is to improve our knowledge on what research has been conducted in the past and explore if it reflects the high-risk priority groups, relevant settings, acknowledges different suicidal behaviors and commonly used suicide methods. Such stock-taking exercise can provide high-level data on our collective research activity and how this aligns with the national suicide prevention agenda.

This in mind, our research group conducted a study over two periods, one in 2006 and one in 2017, which examined where Australia’s suicide-related research efforts have been focused, and whether these have shifted [14–16]. In both cases, we considered suicide-related research that was published and funded over the prior 8-year period between 1999 and 2006 and between 2010 and 2017, and classified it according to a pre-determined framework that categorizes relevant journal articles and grants/fellowships by research type, suicidal behavior, suicide method, target group and research setting.

The current paper uses data from the two time periods and describes how the profile of Australian suicide-related research has changed over time in regard to the emphasis given to different types of suicidal behaviors, suicide methods, target groups and research settings. We made the assumption that published research articles and funded grants/fellowships provide an indication of current research priorities in each of the time periods, and that shifts in the emphasis on these offer insights into trends over time.

Previously we have published a brief report that considered one aspect of suicide-related research only—the type of study [17]. We found that the overwhelming majority (57% in 1999–2006; 60% in 2010–2017) of journal articles described epidemiological studies, and that intervention studies were far less commonly reported (18% in 1999–2006; 14% in 2010–2017). Funding for intervention studies had dropped over the two periods, accounting for 52% of grants/fellowships in 1999–2006 and 30% in 2010–2017, while funding for epidemiological studies increased from 22 to 34%, respectively. These findings were used to inform the funding priorities of the new National Suicide Prevention Research Fund, which is administered by Suicide Prevention Australia. We believe that the data of this current paper can similarly be used to inform the development of a national suicide prevention research agenda and offer guidance on areas that warrant further research attention [16].

## Methods

### Defining and classifying suicide-related research

We defined suicide-related research as “… [involving] activities which collect new data or carry out some novel analysis of existing data, and which pertain to suicide prevention but may not necessarily involve evaluation of suicide prevention initiatives” ([15]; p. 6).

We classified suicide-related research using a framework that captured five overarching categories: research type; suicidal behavior; suicide method; target group; and research setting. Each category was divided into a number of sub-categories which are listed in Tables 1, 2, 3, 4. The framework was developed in 2006 by a group of experts and researchers in the field of suicide prevention and it was kept identical for both periods of data collection to allow for comparisons over time. As noted above, data on research type were presented in a brief report [17]. The focus of the current paper is on the categories of suicidal behavior, suicide method, target group and research setting.

**Table 1:** Research priorities by suicide behavior

Suicide behavior	Articles^a^						Grants/fellowships^a^					
	1999–2006		2010–2017		2 sample prop. test		1999–2006		2010**–**2017		2 sample prop. test	
	#	%	#	%	z-value	p(z)	#	%	#	%	z-value	p(z)
Suicide	100.0	41.6	237.8	56.9	*3.8*	< *0.001*	7.8	41.2	19.5	57.4	1.1	0.258
Attempted suicide	89.5	37.2	95.8	22.9	− *3.9*	< *0.001*	8.3	43.8	7	20.6	− 1.8	0.074
Suicidal thoughts	48.5	20.2	76.7	18.3	− 0.6	0.550	2.8	14.9	7.5	22.1	0.6	0.526
Other suicidal behavior	2.3	1	7.7	1.8	0.8	0.418	0	0	0	0	–	–
Total	240	100	418	100			19	100	34	100		

Test results highlighted in italics mean a significant in the variable of relevance change over time

^a^The number of grants/fellowships and articles may not be integer due to the weighting applied in the coding

**Table 2:** Research priorities by suicide method

Suicide methods	Articles^a^					
	1999–2006		2010–2017		2 sample prop. test	
	#	%	#	%	z-value	p(z)
Poisoning by drugs	20.0	54.1	19.1	34.1	− 1.91	0.056
Poisoning by other (including by gases and vapours)	2.8	7.7	4.8	8.5	0.16	0.877
Hanging (incl. strangulation and suffocation)	3.8	10.4	7.9	14.2	0.54	0.590
Firearms (incl. explosives)	4.3	11.7	4.4	7.9	− 0.61	0.539
Drowning	1.0	2.7	0.8	1.4	− 0.45	0.655
Jumping from a high place	1.5	4.1	6.1	10.9	1.17	0.242
Jumping or lying before a moving object	0.0	0.0	6.5	11.6		*0.044*^b^
Other	3.5	9.5	6.3	11.2	0.26	0.794
Total	37.0	100.0	56			

Test results highlighted in italics mean a significant in the variable of relevance change over time

^a^The number of grants/fellowships and articles may not be integer due to the weighting applied in the coding

^b^Fisher’s exact test

**Table 3:** Research priorities by target group

Target groups	Articles^a^						Grants/fellowships^a^					
	1999–2006		2010–2017		2 sample prop. test		1999–2006		2010–2017		2 sample prop. test	
	#	%	#	%	Z	p(z)	#	%	#	%	Z	p(z)
Young people (aged 24 or less)	58.1	27.8	57.0	18.0	− *2.7*	*0.008*	12.7	48.7	13.8	49.4	0.1	0.959
Adults (aged 25–64)	5.4	2.6	29.0	9.1	*3.0*	*0.003*	0.0	0.0	0.0	0.0	–	–
Older people (aged 65 or more)	12.3	5.9	17.0	5.4	− 0.2	0.807	2.0	7.7	0.0	0.0		0.491^b^
Indigenous people	8.3	4.0	15.0	4.7	0.4	0.702	0.0	0.0	2.3	8.3		0.242^b^
People from culturally or linguistically diverse backgrounds	1.0	0.5	2.0	0.6	0.2	0.880	0.0	0.0	0.0	0.0	–	–
People in rural and remote areas	7.8	3.7	10.0	3.2	− 0.3	0.757	1.3	5.1	2.3	8.3	0.5	0.640
People bereaved by suicide	2.0	1.0	19.0	6.0	*2.9*	*0.004*	0.0	0.0	1.0	3.6		1.0^b^
People who are gay, lesbian, bisexual, transgender, intersex	2.5	1.2	5.0	1.6	0.4	0.706	0.3	1.3	0.0	0.0		0.491^b^
People with mental health problems	22.7	10.8	37.0	11.7	0.3	0.750	2.8	10.9	2.5	8.9	− 0.3	0.805
People with substance use problems	11.0	5.3	12.0	3.8	− 0.8	0.412	1.0	3.8	1.0	3.6	0.0	0.969
People with physical health problems	6.5	3.1	12.0	3.8	0.4	0.670	0.0	0.0	0.0	0.0	–	–
People who have attempted suicide	37.7	18.0	21.0	6.6	− *4.1*	< *0.001*	3.8	14.7	1.5	5.4	− 1.2	0.252
Offenders	9.5	4.5	6.0	1.9	− 1.7	0.084	1.5	5.8	1.0	3.6	− 0.4	0.702
Men	7.1	3.4	15.0	4.7	0.7	0.466	0.5	1.9	1.5	5.4	0.7	0.497
Women	3.0	1.4	8.0	2.5	0.9	0.385	0.0	0.0	0.0	0.0	–	–
Current or ex-serving military personnel	0.0	0.0	1.0	0.3		1.0^b^	0.0	0.0	0.0	0.0	–	–
Other	14.2	6.8	51.0	16.1	*3.2*	*0.002*	0.0	0.0	1.0	3.6		1.0^b^
Total	208.9	100.0	317.0	100.0			26.0	100.0	28.0	100.0		

Test results highlighted in italics mean a significant in the variable of relevance change over time

^a^The number of grants/fellowships and articles may not be integer due to the weighting applied in the coding

^b^Fisher’s exact test

**Table 4:** Research priorities by research setting

Research settings	Articles^a^						Grants/fellowships^a^					
	1999–2006		2010–2017		2 sample prop. test		1999–2006		2010–2017		2 sample prop. test	
	#	%	#	%	z-value	p(z)	#	%	#	%	z-value	p(z)
Communities	22.5	17.0	20.0	10.1	− 1.83	0.067	3.0	18.8	11.0	35.5	1.19	0.236
Schools	10.5	8.0	7.0	3.5	− 1.79	0.074	3.0	18.8	5.0	16.1	− 0.23	0.815
Tertiary institutions	5.0	3.8	6.0	3.0	− 0.40	0.691	0.0	0.0	1.0	3.2		1.0^b^
Prisons	11.0	8.3	6.0	3.0	− *2.14*	*0.032*	1.0	6.3	0.0	0.0		0.340^b^
Workplaces	0.0	0.0	33.0	16.7		< *0.001*^b^	0.0	0.0	1.0	3.2		1.0^b^
Primary care settings (e.g. general practice)	9.5	7.2	8.0	4.0	− 1.27	0.203	1.0	6.3	1.0	3.2	− 0.50	0.618
Emergency departments	15.0	11.4	15.0	7.6	− 1.18	0.240	1.0	6.3	0.5	1.6	− 0.87	0.386
Mental health service settings	16.0	12.1	32.0	16.2	1.04	0.301	3.0	18.8	4.0	12.9	− 0.54	0.590
Other health services	41.5	31.4	32.0	16.2	− *3.25*	*0.001*	4.0	25.0	0.0	0.0		*0.019*^b^
Other settings	1.0	0.8	39.0	19.7	*5.15*	< *0.001*	0.0	0.0	7.5	24.2		0.089^b^
Total	132.0	100.0	198.0	100.0			16.0	100.0	31.0	100.0		

Test results highlighted in italics mean a significant in the variable of relevance change over time

^a^The number of grants/fellowships and articles may not be integer due to the weighting applied in the coding

^b^Fisher’s exact test

### Data collection

To identify suicide-related journal articles in each time period several international databases were systematically searched using the terms “suicide* OR self harm OR suicide* attempt* AND Australia”. In 2006, we searched Medline, PsychInfo and CINHAL, and in 2017 we added AUSTInfo and ISI Web of Science. We included peer-reviewed articles that described studies that met the criteria for suicide-related research as defined by the framework. We excluded articles that focused on euthanasia (assisted dying), did not include a full abstract, did not involve primary research, were systematic or narrative reviews or evidence-based commentaries, did not have a first author with an Australian address, and/or reported research conducted outside Australia. All records identified in the searches were exported into an Excel Spreadsheet for screening and all eligible records were exported into SPSS for coding against categories.

Following our search terms, we identified a total of 373 records (excluding duplicates) in 2006 and their abstracts were screened for inclusion. 110 articles were excluded as they did not fit the selection criteria, mostly because their primary focus was not on suicidal behavior. The remaining 263 articles were eligible and were included in data coding. In 2017, a total of 555 records were identified and after exclusion of 131 records based on abstract screening against our selection criteria, 424 records remained for coding.

Information on funded grants/fellowships was retrieved from the respective website repositories of the Australian granting bodies that provide the majority of funding for suicide-related research. In 2006, these were the National Health and Medical Research Council, the Australian Research Council and Australian Rotary Health. In 2017, a fourth body—the Society for Mental Health Research—was added. We included grants/fellowships if they had a start date within the two relevant periods (i.e., between 1999 and 2006 or between 2010 and 2017), had a primary focus on suicide, and were conducted in Australia. We also recorded the amount of funding provided for each grant/fellowship.

### Data coding and analysis

Each abstract was examined and classified by a single team member to the predetermined coding framework. Where necessary consultations were held with another team member. To ensure consistency of coding 14 abstracts were randomly selected and independently coded to the framework categories by two team members. Differences in coding were resolved by cross reviewing of independent coding results and discussions between the two coders. The full dataset was then divided in halves, and the same two coders separately coded each half of abstracts. Where more information for coding was required the full-text document was sourced.

We extracted relevant descriptive information from journal article abstracts and grant/fellowship summaries. Each abstract and grant/fellowship summary was then coded against the classifications in each category of the framework. Where articles or grants/fellowships pertained to more than one classification, codes were weighted by summing them up to one to avoid double counting. One member of our team coded all articles and grants/fellowships in the first period and two members did so in the second period. Care was taken to ensure fidelity of coding to the classification (e.g., with recourse to the team leader to resolve issues in both periods, and double-coding and cross-checking of some articles and grants/fellowships in the second period). Where articles and grants/fellowships did not have sufficient information for coding to classifications, this resulted in a denominator lower than the total number of articles or grants/fellowships.

Data were analyzed in SPSS and two-sample tests of proportion were conducted in Stata14 for each sub-category to test for changes over time. Where a category showed as zero articles or grants/fellowships in either period we used Fisher’s exact test for comparisons across time.

## Results

Notwithstanding the fact that we included some additional databases in our search for suicide-related articles and an additional funding body for grants/fellowships in 2010–2017, it appears that suicide-related research has grown over time. While the total number of grants remained stable at 36 funded grants with a focus on suicide-related research the total amount of funding nearly doubled, rising from just under 5.8 million Australian dollars to 10.5 million. The number of published articles increased by 62% from 262 in 1999–2006 to 424 in 2010–2017.

### Suicidal behavior

Table 1 shows the relative proportions of articles and grants/fellowships in each period that focused on suicide, suicide attempts or suicidal thoughts as their outcome of interest.

The type of suicidal behavior could be determined for 418 articles in 2010–2017 and 240 articles in 1999–2006. In 2010–2017, the majority of suicide-related articles focussed on suicide (57%), with a smaller proportion focusing on suicide attempts (23%), and a still smaller proportion on suicidal thoughts (18%). The order was the same in 1999–2006, but the proportions were different (with 42% focusing on suicide, 37% on attempted suicide and 20% on suicidal thoughts). The increase in articles focusing on suicide was statistically significant (z-value = 3.78; p-value < 0.001), as was the decrease in articles focusing on suicide attempts (z-value = − 3.93; p-value < 0.001).

Suicidal behavior that was the focus of 34 grants/fellowships in 2010–2017 and 19 in 1999–2006. In 2010–2017, suicide was the outcome of interest in 57% of all funded grants/fellowships, with relatively lesser funding emphasis given to suicide attempts (21%) and suicidal thoughts (22%). This pattern contrasts with that of 1999–2006 where similar proportions of grants/fellowships focused on suicide and suicide attempts (41% and 44%, respectively), leaving only 15% focusing on suicidal thoughts. Differences in proportions across the two periods were not statistically significant.

### Suicide methods

Table 2 summarises the relative proportions of articles that were concerned with each of the eight suicide methods in our classification, by data collection period. The grant/fellowship summaries did not provide sufficient information to conduct equivalent cross-period funding analyses.

Relatively few articles in either period focused on a specific suicide method (56 of 424 in 2010–2017 and 37 of 262 in 1999–2006). In 2010–2017, 34% of the 56 articles were accounted for by those that examined poisoning by drugs. This was followed by articles on hanging (14%), jumping or lying before a moving object (12%) and jumping from a high place (11%). Compared with 1999–2006, this represented an apparent decrease in articles on poisoning by drugs (from 54%) and increase in articles on hanging and jumping from a high place (from 10% and 4%, respectively). Articles focusing on jumping or lying before a moving object represented a new research interest, since there were none in 1999–2006 (Fisher’s exact = 0.044). Comparatively lesser emphasis was given to self-poisoning by means other than drugs, suicide by drowning and by using firearms, and this was consistent over time.

### Target groups

Table 3 shows the spread of articles and grants/fellowships accounted for by research into the 17 target groups in our classification for the two data collection periods.

The target group could be identified for 317 articles in 2010–2017 and 209 articles in 1999–2006. In 2010–2017, all target groups identified in our classification featured in published articles, suggesting that at least some research was being devoted to each of them. Target groups that accounted for relatively larger proportions of articles were young people (aged 24 or less; 18%), people with mental health problems (12%), adults (aged 25–64; 9%); people who have attempted suicide (7%), and people bereaved by suicide (6%). ‘Other’ target groups accounted for 16% of articles. Comparing this picture with that from 1999 to 2006 shows that the emphasis on young people and people who have attempted suicide significantly decreased [from 28% (z = − 2.7, p = 0.008) and 18% (z = − 4.1, p < 0.001), respectively]. By contrast, there were significant increases in the proportion of published articles focussing on adults [from 3% (z = 3.0, p = 0.003)], people bereaved by suicide [from 1% (z = 2.9, p = 0.004)], and ‘other’ target groups [from 7% (z = 3.2, p = 0.002)]. The increase in listing ‘other’ target groups was due to naming a larger number of specific groups which did not fit in the pre-determined classifications in 2017. In the 2006 data the main groups in the ‘other’ category were migrants/asylum seekers/detention centres, victims of sexual abuse, and people in metropolitan areas. In the 2017 data, the main groups had shifted to health professionals and a large variety of workforce groups.

The target group could be ascertained for 28 grants/fellowships in 2010–2017 and 26 grants/fellowships in 1999–2006. In 2010–2017, almost half (49%) of all grants/fellowships were accounted for by research focusing on young people. Other notable target groups in that period were people with mental health problems (9%), Indigenous people (8%), and people in rural and remote areas (8%). In 1999–2006, the same proportion of all grants/fellowships focused on young people (49%), and similar proportions focused on people with mental health problems (11%) and people in rural and remote areas (5%). Importantly, however, none focused on Indigenous people in the earlier period, and considerably more focused on people who have attempted suicide (15% compared with 5%). Differences in proportions across the two periods were not statistically significant.

### Research settings

Table 4 provides information on the settings where the research described in articles and funded through grants/fellowships took place for both periods.

The research setting could be identified in 198 articles in 2010–2017 and 132 articles in 1999–2006. In 2010–2017, sizeable proportions of suicide-related articles described research that occurred in workplaces (17%), mental health service settings (16%), other health service settings (16%) and settings classified as ‘other’ (20%). The proportion of articles reporting on studies conducted in other health services dropped significantly [from 31% in 1999–2006 (z = − 3.25, p = 0.001)]. The same was true for articles reporting on studies set in prisons, which reduced from 8 to 3% (z = − 2.14, p = 0.032). By contrast, the proportion of articles representing workplace-based studies increased from a baseline of zero (Fisher’s exact = 0.0), and the proportion of articles reporting on studies conducted in ‘other’ settings increased from 1% (z = 5.15, p < 0.001). The increase on the listing of other research settings has two reasons. First, almost half of the articles included in the ‘other’ category related to online settings, and these were nascent in the earlier period. Second, the variety and specificity in settings had increased which meant these fitted nowhere else in the coding framework. These mainly included specific workforce industries and were a once only occurrence.

Information on research setting was available for 31 grants/fellowships in 2010–2017 and 16 grants/fellowships in 1999–2006. In 2010–2017, 36% of grants/fellowships focussed on research in community settings (up from 19% in 1999–2006). Further funding emphasis was given to settings classified as ‘other’ (24%), schools (16%) and mental health services (13%). Grants/fellowships that involved research in ‘other settings’ increased from zero in 1999–2006 to 24% in 2010–2017, with most grants/fellowships in this category (4.5 of 7.5) accounted for by social media or online settings. By contrast, there was a significant decrease in grants/fellowships that involved studies conducted in health services (from 25% in 1999–2006 to zero in 2010–2017; Fisher’s exact = 0.019).

## Discussion

It is encouraging to see that overall funding for suicide-related research in Australia has increased over the two study periods (1999–2006 to 2010–2017). Similarly, the number of published articles increased substantially over the 20-year period. Both indicate an acknowledgment by researchers and funding bodies alike that suicide is a major public health issue and warrants increased research attention [16]. However, a growing momentum in suicide-related research does not by itself mean a focus on the most pressing issues in suicide prevention [18].

We compared study data from two periods (1999–2006 and 2010–2017) and analysed how suicide-related research has shifted over these periods. Our study found that research into suicide featured more strongly than research into attempted suicide and suicidal thoughts in articles and grants/fellowships from both time periods. In the second time period there was a decreased emphasis on attempted suicide in both research articles and grants/fellowships and a further increase in research articles and grant/fellowships on suicide. Yet, suicide attempts are a significant predictor of suicide, and focusing on those who have made suicide attempts in the past and enhancing our understanding of what drives attempts may help to avert future suicide [19]. Suicide attempts also constitute a significant issue in their own right and research into their prevention and management is important. In recent times, considerable policy attention has been given to ensuring that people who attempted suicide are well-supported after the event. In Australia, the Way Back Support Service, which is delivered to people who have been admitted to a hospital following a suicide attempt or people experiencing a suicide crisis is one good example [20]. A qualitative evaluation of this service is currently underway [21].

The decrease in research on suicide attempts may be linked to an earlier study highlighting the continued prioritization of epidemiological research over intervention studies [17]. Research on suicide often uses epidemiological data from national data registers to better understand suicide behavior while intervention studies are more likely to focus on prevention of suicide attempts and suicidal thoughts involving participants. While this may provide a possible explanation, it is not an acceptable reason. More research effort should be invested into the group of suicide attempters as this group is in part known to health services and finding effective interventions that reduce their risk for repeated attempt are crucial and a knowledge gap that needs closing [2]. This sentiment is confirmed by findings published from a key expert and stakeholder survey on ratings for future priorities in the field [15].

With respect to the emphasis given to particular suicide methods, our study provides limited insight. We were not able to extract information on suicide methods from the grant/fellowship abstracts, and only a relatively small number of the articles specifically dealt with particular methods. In the majority of cases, this was probably because the articles were about suicide and its prevention in general, and not about the particular methods that individuals may have chosen. Where suicide methods were a focus, there appeared to be some shift over time, with a reduced emphasis on poisoning and an increased emphasis on suicides by jumping (from heights and in front of moving objects). Some of the latter work may align with increased interest in restricting access to means at so-called ‘suicide hotspots’ (e.g., bridges and cliffs, railway tracks) [22, 23]. Internationally, there is recognition that interventions like barriers at these sites are effective in reducing suicide, and in Australia funding has been provided to secure a number of sites [24, 25]. Despite the fact that hanging is the most commonly used lethal method in Australia we did not see this reflected in the research activities. Given its dominance it may be of benefit to encourage research on how interventions could target making choosing hanging as a method less accessible. Suicide related research into specific target groups shows both, areas of consistent research activity as well as new research priorities developing over time. The good news is that young people and people with mental health problems, which are high-risk populations, have seen consistent research activity. Three other groups which are also defined as high priority groups by the Government also show increased research activity. These are Indigenous people, men and those bereaved by suicide. For Indigenous people suicide rates are particularly high [1, 26], their suicidal behaviors are poorly understood, and effective interventions are lacking [27]. The same can be said for male specific interventions [6]. The increased focus on people bereaved by suicide reflects increased emphasis on the exposure to and the impact of suicide on the wider community [28–31]. While increasing research in these population groups is encouraging, overall the activity still remains low. However, there are still target groups such as the LGBTIQA community, which despite being highlighted as a high-risk group has not yet received adequate research attention, therefore risking a widening of the knowledge gap for this group.

The settings in which suicide-related research was conducted showed consistency as well as new developments. Research in community settings, schools and mental health services were a consistent focus over time. The consistent emphasis is not surprising because these settings are often the sites of universal, selective and indicated suicide prevention activities and they are consistent with addressing some of the priority areas outlined in the 5th National Mental Health and Suicide Prevention Plan [32]. Others came to the fore in the latter period, particularly workplaces and ‘other’ settings (which often included online environments). The newer settings may reflect a desire to understand the physical and virtual locations where people who may be at risk of suicide congregate and to capitalise on them as sites for suicide prevention [33, 34].

We are conscious that our study has some limitations, particularly in relation to the grants/fellowships. We only included information on grants/fellowships from the main academic funding bodies relevant to suicide-related research. While there are other important non-for-profit and philanthropic funding bodies, we were restricted to those data records that offer a systematic and publicly available reporting system for funded projects and allowed data extraction. The lack of detail in available grant/fellowship summaries may have meant that we excluded some grants/fellowships that did in fact involve suicide-related research. The lack of detail in both the grant/fellowship summaries and publication abstracts may have also introduced some classification errors. Further, the lack of statistically significant differences in comparisons over time may be explained by the relatively low numbers of articles and grants/fellowships in some categories.

Notwithstanding these limitations, this study provides an overview of where suicide-related research priorities lie and how these have shifted over time. This stock-taking exercise provides high level data on research activity and indicates how research aligns with the national suicide prevention agenda. We encourage funding agencies and suicide prevention researchers to utilize the presented data to further the conversation on whether research priorities need to be broadened or shifted [35].

Of course, additional data sources should also be consulted to set future priorities. Expert and stakeholder views can be used to inform a discussion on emerging and pressing issues in current suicide prevention. This is why we also conducted complementary work eliciting the opinions of those who fund, use and conduct suicide-related research, as well as those with lived experience of suicidal behavior. We flagged some of these findings in our brief report on the priority given to particular types of suicide-related research [17], and throughout this paper to inform our interpretation of findings. Other important objective metrics for gauging priority are the relative risk and population attributable risk of suicide and suicidal behaviors for particular target groups. In the Australian context, we highlight the need for encouraging and supporting research into people who have history of suicide attempt (lived experiences), Indigenous people, men, and the LGBTIQA community [9, 32]. These groups have been highlighted in various sources as high risk and therefore priority groups for research since 2010, yet our study found these groups to be underrepresented in research activity. We note though that this observation is not to ignore other high-risk minority groups (such as prisoners and elderly people) which have yet to be more fully recognised as such.

## Conclusion

Suicide-related research has increased in Australia over the last decade, in terms of both inputs (funding) and outputs (publications). In some cases, research priorities appear to have been fairly consistent over time and in others new foci have emerged. To some extent, these patterns may be a reflection of changes in what the broader suicide prevention field views as important. In other cases, however, it is likely that the research can drive policy and practice change. For this reason, prioritizing research that is focused on areas with high population level risk as well as those that are recognized by specialists and key stakeholders in the field is crucial. We encourage funding agencies and suicide prevention researchers to utilize the presented data to further the conversation on where future research priorities should lie.

## Data Availability

The search strategies as well as output tables are included in this paper. Further data reports on the two studies can be requested through the authors.
